# ER-Phagy: A New Regulator of ER Homeostasis

**DOI:** 10.3389/fcell.2021.684526

**Published:** 2021-07-09

**Authors:** Ming Yang, Shilu Luo, Xi Wang, Chenrui Li, Jinfei Yang, Xuejing Zhu, Li Xiao, Lin Sun

**Affiliations:** ^1^Department of Nephrology, The Second Xiangya Hospital, Central South University, Changsha, China; ^2^Hunan Key Laboratory of Kidney Disease and Blood Purification, Changsha, China; ^3^Department of Nutrition, Xiangya Hospital, Central South University, Changsha, China

**Keywords:** endoplasmic reticulum (ER), unfolded protein response (UPR), ER-phagy, autophagy, ERAD

## Abstract

The endoplasmic reticulum (ER) is one of the most important cellular organelles and is essential for cell homeostasis. Upon external stimulation, ER stress induces the unfolded protein response (UPR) and ER-associated degradation (ERAD) to maintain ER homeostasis. However, persistent ER stress can lead to cell damage. ER-phagy is a selective form of autophagy that ensures the timely removal of damaged ER, thereby protecting cells from damage caused by excessive ER stress. As ER-phagy is a newly identified form of autophagy, many receptor-mediated ER-phagy pathways have been discovered in recent years. In this review, we summarize our understanding of the maintenance of ER homeostasis and describe the receptors identified to date. Finally, the relationships between ER-phagy and diseases are also discussed.

## Introduction

The endoplasmic reticulum (ER) is one of the most important organelles that play a role in various cellular processes, such as the synthesis, transport, and posttranslational modification of proteins and the storage of intracellular calcium (Oakes and Papa, 2015; Marciniak, 2017; Dikic, 2018). In cells, almost all secretory and membranous proteins must be folded and assembled in the ER, and the abundance of protein chaperones in the ER and the homeostasis of the ER microenvironment ensure that proteins are folded and processed correctly (Rashid et al., 2015). However, the ER is particularly sensitive to internal and external stimuli; when faced with environmental and pathological conditions such as high glucose (Chen et al., 2018) and oxidative stress (Dandekar et al., 2015), ER homeostasis is disrupted, resulting in the accumulation of unfolded proteins in the ER and the subsequent induction of ER stress. The accumulation of excess unfolded proteins in the ER lumen causes ER dysfunction. Common pathways are activated to counteract ER stress and restore the functions of the ER, including the unfolded protein response (UPR) and ER-associated degradation (ERAD) (Wilkinson, 2019). A recently proposed phenomenon, ER-phagy, has also been shown to be involved in the maintenance of ER homeostasis. ER-phagy is a form of selective autophagy that is mainly mediated by specific ER-phagy receptors, proteins that reside in the ER or cytosol that are recruited to the ER membrane, thereby controlling the time point of ER degradation; ER-phagy receptors also have the ability to interact with the autophagy-related protein LC3/ATG8 through its LC3-interacting region (LIR) (Loi et al., 2018; Song et al., 2018). Abnormal ER-phagy prevents the degradation of dysfunctional ER, eventually causing a number of diseases (Islam et al., 2018; Cai et al., 2019a). Recently, some new ER-phagy receptors have been discovered, but research in the field of ER-phagy is still emerging. Moreover, although several studies have described the relationship between ER-phagy receptors and diseases, these associations are only speculative at this point. Therefore, in this review, we will focus on the maintenance of ER homeostasis, summarize the identified ER-phagy receptors, and, finally, discuss the potential relationships between abnormal ER-phagy and disease.

### Unfolded Protein Response

**FIGURE 1:**
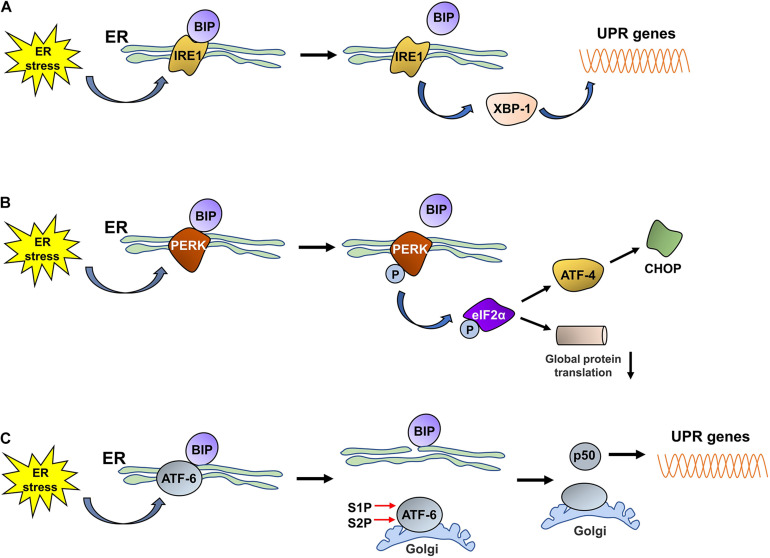
The three UPR pathways activated by ER stress.**(A)** Under ER stress conditions, IRE1 dissociates from BiP and is subsequently activated. Then, activated IRE1 promotes the translation of XBP1, and increased XBP1 levels promote the expression of UPR proteins. **(B)** Under ER stress conditions, PERK dissociates from BiP and is then self-phosphorylated; phosphorylated PERK induces the phosphorylation of eIF2α. Phosphorylated eIF2α decreases the rate of global protein translation and upregulates the expression of ATF4, which promotes CHOP expression. **(C)** ER stress promotes the dissociation of ATF6 from BiP. ATF6 is then translocated to the Golgi, where it is cleaved by S1P/S2P, and the cytosolic p50 fragment is released. p50 upregulates the expression of XBP1; subsequently, the expression of UPR-related proteins is also increased.

The UPR is an adaptive response that is induced under ER stress conditions; signal sensors in the ER detect disturbances in the ER lumen and activate the downstream signaling cascades of the UPR to re-establish ER homeostasis by reducing protein synthesis, increasing protein folding, and accelerating misfolded protein degradation (Bhat et al., 2017; Smith and Wilkinson, 2017). The UPR is activated by three unique ER stress sensors: pancreatic ER kinase-like ER kinase (PERK), inositol-requiring enzyme 1 (IRE1), and activating transcription factor 6 (ATF6). In a normally functioning ER, PERK, IRE1, and ATF6 are bound to the chaperone BiP/GRP78 in the ER lumen. Upon ER stress, the proteins (PERK, IRE1, and ATF6) dissociate from BiP/GRP78 and exert their biological effects on downstream molecules to relieve the stress (Figure 1).

### IRE1

Inositol-requiring enzyme 1 is an ER-resident transmembrane protein that has both kinase and endoribonuclease activity and is the most evolutionarily conserved UPR sensor (Acosta-Alvear et al., 2018; Mitra and Ryoo, 2019). IRE1 has two homologs, IRE1α and IRE1β, in the murine and human genomes (Ni et al., 2018). Under ER stress conditions, the unfolded proteins that accumulate in the ER lumen bind to BiP to induce its dissociation from IRE1, which is activated through dimerization, autophosphorylation, and further oligomerization. Then, activated IRE1 exerts RNase activity and splices the mRNA encoding X-box binding protein 1 (XBP1) to promote the translation of XBP1 (Yang et al., 2016; Adams et al., 2019). Increased XBP1 expression promotes the expression of chaperones and ERAD-related proteins to reduce or stop the ER stress response and thus restore cellular homeostasis.

### PERK

Pancreatic ER kinase-like ER kinase is a transmembrane protein that is widely expressed throughout the body and contains an N-end stress sensing domain and a cytosolic kinase domain (Lebeaupin et al., 2018; Hughes and Mallucci, 2019). Under stable ER conditions, PERK binds to BiP to form an inactive complex. Upon ER stress, PERK is activated by oligomerization and self-phosphorylation, which enables the phosphorylation of a variety of PERK substrates, including eukaryotic translation initiation factor 2 (eIF2α), NF-E2-related factor 2 (Nrf2), forkhead box O (FOXO) proteins, and the second messenger diacyglycerol (DAG) (Pytel et al., 2016; Cubillos-Ruiz et al., 2017). The released BiP induces the autophosphorylation of PERK; the activated PERK then phosphorylates eIF2α, the main substrate of PERK, which inhibits the assembly of the eIF2-GTP-Met-tRNA ternary complex, thereby decreasing the rate of global protein translation to reduce the burden of protein folding in the ER and ensure the alleviation of ER stress (Oakes, 2020). Although short interruptions in protein translation, which provide cells extra time to process stored proteins, are beneficial to cells under ER stress, a long period of protein translation interruption is detrimental to cell survival. Phosphorylated eIF2α terminates the translation of some mRNAs by blocking 80S ribosome assembly, while increasing the translation of other mRNAs with upstream open reading frames within their 5′ untranslated regions, such as ATF4 (Lebeaupin et al., 2018). ATF4 is a transcription factor that activates UPR target genes associated with protein folding and apoptosis (Wortel et al., 2017; Kasai et al., 2019) and regulates the expression of C/EBP homologous protein (CHOP) (Averous et al., 2004). In addition to the PERK-eIF2α-ATF4 pathway, the activated PERK-mediated phosphorylation of the Nrf2 and FOXO proteins plays a fundamental role in regulating cellular metabolic adaptation under ER stress conditions. The phosphorylation of Nrf2 in response to ER stress promotes its release from its repressor, Kelch-like enoyl-CoA hydratase (ECH)-associated protein 1 (KEAP1); Nrf2 subsequently translocates to the nucleus and induces the expression of multiple antioxidant proteins that alleviate the effects of stress-induced reactive oxygen species (ROS) and facilitate adaptation to oxidative stress. Similarly, PERK activates FOXO proteins, which exert a negative regulatory effect on AKT activity, thus switching the metabolic program of stressed cells from anabolism to catabolism (Cubillos-Ruiz et al., 2017; Nam and Jeon, 2019). Thus, the activation of PERK plays an important role in maintaining ER homeostasis and promoting the survival of stressed cells.

### ATF6

Activating transcription factor 6 is a type II transmembrane protein that resides in the ER and is present in two isoforms, ATF6 alpha and ATF6 beta. ATF6 contains a basic leucine zipper-binding domain (bZIP) within its cytosolic domain (Cubillos-Ruiz et al., 2017; Moon et al., 2018). Similar to IRE1 and PERK, ATF6 binds to GRP78/BiP, thereby maintaining the complex in an inactivated state. Upon ER stress, ATF6 is released from GRP78/BiP and translocates to the Golgi apparatus *via* coat protein complex II (COPII)-coated vesicles; ATF6 thereafter is cleaved, and the cytosolic p50 fragment is generated in the presence of the Golgi enzymes site 1 protease (S1P) and S2P (Lin et al., 2019). The cytosolic p50 fragment is a transcription factor that regulates the expression of XBP1 and the genes needed for ERAD, thereby promoting the ability of the ER to address ER stress (Endres and Reinhardt, 2013). In addition, the cytosolic p50 fragment also regulates the expression of sterol regulatory element-binding proteins (SREBPs) (Lin et al., 2019) and ER expansion (Bommiasamy et al., 2009).

In summary, the UPR is activated by the accumulation of misfolded proteins in the ER and relieves ER stress by slowing the translation of proteins, thereby reducing the protein load in the ER. Moreover, the UPR also upregulates the expression of chaperone proteins that promote the folding or removal of misfolded proteins. However, sustained ER stress transforms the UPR from a protective pathway to a proapoptotic pathway, which is the pathological basis of many diseases.

### ERAD

In addition to the UPR, ERAD is a protective mechanism that is activated in cells in response to ER stress. ERAD, which denotes “ER-associated protein degradation,” is a pathway that prevents the accumulation of misfolded proteins in the ER; during this process, misfolded polypeptides are transported back to the cytosol and degraded by the ubiquitin-proteasome system (Mehrtash and Hochstrasser, 2019). Briefly, a protein substrate in the ER that is targeted for degradation is recognized by specific proteins and then transferred to the cytoplasmic side of the ER, where it is ubiquitinated by a ubiquitin ligase. Finally, the ubiquitinated substrate is released from the ER into the cytoplasm *via* an ATP-dependent pathway and degraded by the proteasome (Ruggiano et al., 2014). ERAD not only is a process dedicated to ER protein quality control but also controls the turnover of specific proteins to achieve certain physiological states (Ruggiano et al., 2014). ERAD regulates the cellular contents of some key proteins involved in lipid biosynthesis and calcium homeostasis, such as 3-hydroxy-3-methyl-glutaryl acetyl coenzyme-A reductase (HMGR), a rate-limiting enzyme involved in the synthesis of cholesterol (Erffelinck and Goossens, 2018; Wangeline and Hampton, 2018). The degradation of HMGR by ERAD results in reduced flux through the sterol biosynthetic pathway and in the reestablishment of membrane lipid homeostasis (Fernandez et al., 2015). In addition to ERAD, ER-to-lysosome-associated degradation (ERLAD) also controls ER quality and responds to ER stress (Fregno and Molinari, 2019). ERLAD is responsible for the removal of misfolded proteins that are too large for ERAD degradation and functions by transferring these proteins to lysosomes for degradation (Almanza et al., 2019; De Leonibus et al., 2019; Fregno and Molinari, 2019). The precise molecular mechanism of ERLAD has not been thoroughly studied, but this process plays an important role in alleviating ER stress and maintaining the intracellular balance, indicating that it deserves further study.

## ER-Phagy Receptors

**FIGURE 2:**
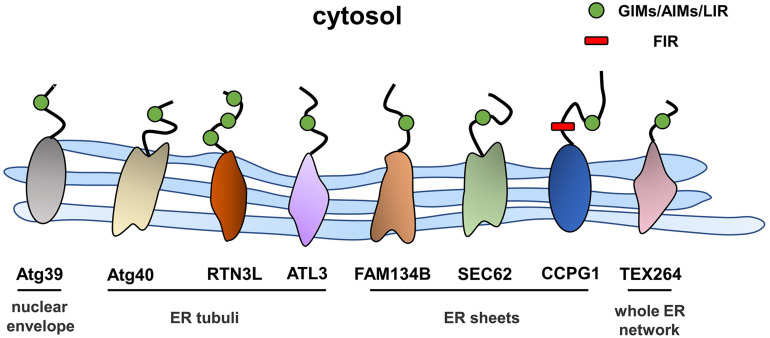
The structures and locations of ER-phagy receptors identified in yeast and mammals.

Autophagy is a process in which excess proteins or organelles are encapsulated by vesicles that fuse with lysosomes to form autophagic lysosomes, which then degrades the encapsulated content (Glick et al., 2010; Shibutani et al., 2015). Autophagy is divided into different subtypes depending on the degradation content (Johansen and Lamark, 2011; Lamark et al., 2017). ER stress is an initiator of autophagy, and ER-phagy represents a subclass of ER stress-mediated autophagy, which degrades spent proteins, excess proteins, and damaged organelles, while ER-phagy selectively degrades excessive or damaged ER (Song et al., 2018). ER-phagy involves an autophagosome that directly connects to the ER through ER-phagy receptors and degrades excess ER. ER-phagy plays a key role in maintaining cellular homeostasis by eliminating excess ER in a timely manner and preventing cells from being damaged by intense ER stress (Grumati et al., 2018). In the next sections, we will focus on the ER-phagy receptors that have thus far been identified (Figure 2).

## ER-Phagy Receptors in Mammals

### FAM134B

FAM134B, the first identified and best-characterized ER-phagy receptor in mammals, contains an ER protein homology domain (reticulon homology domain, RHD) that promotes the bending of the ER membrane (Bhaskara et al., 2019). In addition, an LC3 interaction domain is present at the C-terminus of FAM134B in the cytoplasm, and the working region (LIR) anchors LC3 or GABARAP to autophagic vesicles. This structure is a prerequisite for FAM134B to function as an ER-phagy receptor, and a variety of factors that regulate the activity of ER-phagy are modulated by FAM134B. Under ER stress conditions, the RHD of FAM134B is phosphorylated by activated CAMK2B, thus enhancing the oligomerization of FAM134B and membrane fragmentation to meet the high demand for ER-phagy (Jiang et al., 2020). The RHD of FAM134B has been shown to have three potential phosphorylation sites, the serine residues S149, S151, and S153, which regulate the oligomerization of FAM134B through their phosphorylation within the RHD and then regulate the scission of the ER during the ER-phagy process; CAMK2B is a putative kinase responsible for the phosphorylation of FAM134B at S151 (Jiang et al., 2020). In addition, calnexin (CNX), the ER-resident lectin chaperone, is involved in the FAM134B-mediated clearance of misfolded proteins. Fregno et al. (2018) were the first to reveal that the ERLAD pathway of alpha1-antitrypsin Z (ATZ) requires CNX and FAM134B. Moreover, Forrester et al. reported that CNX functions as a sensor of misfolded procollagens in the ER lumen and interacts with the ER-phagy receptor FAM134B. In addition, FAM134B binds to the LC3 protein through its LIR region and transports it to lysosomes for degradation (Forrester et al., 2019). Additionally, a recent study found that the nutrient responsive transcription factors TFEB and TFE3 regulate lysosomal biogenesis and control ER-phagy by promoting the expression of FAM134B (Cinque et al., 2020). Furthermore, an N-terminal-truncated isoform of FAM134B, FAM134B-2, has been shown to be involved in starvation-induced ER-phagy. In the starvation state, CCAAT/enhancer binding protein β (C/EBPβ) upregulates the expression of FAM134B-2 and then recruits it to autophagosomes (Kohno et al., 2019). In general, ER stress and the UPR induce ER-phagy, and the resulting excessive activation of ER-phagy induced by FAM134B leads to ER stress and to the UPR. In HeLa cells, the small molecule Z36 upregulated the expression of FAM134B, LC3, and ATG9, which work together to promote ER-phagy as evidenced by an increased number and enlarged volume of autophagosomes. Furthermore, overactivation of ER-phagy leads to increased ER degradation and impaired ER homeostasis, which eventually triggers ER stress and cell death (Liao et al., 2019).

### SEC62

The transporter SEC62 is part of the SEC61/SEC62/SEC63 transport complex of ER transmembrane components that mediates the translocation of polypeptides in the ER lumen (Linxweiler et al., 2017). Additionally, SEC62 contains a conserved LIR at its C-terminus that enables the independent induction of ER-phagy (Fumagalli et al., 2016). During recovery from ER stress induced by cyclopiazonic acid and dithiothreitol, ER-phagy was shown to be induced by the interaction of the SEC62 LIR with LC3 (Fumagalli et al., 2016), and this process was independent of SEC61 and SEC63. Mechanistically, the binding of LC3 binding to SEC62 was activated upon the termination of ER stress, and the excess ER was then consumed by lysosomes. This process involves the endosomal sorting complex that is required for the transport (ESCRT)-III component CHMP4B and the accessory AAA + ATPase VPS4A (Loi et al., 2019; Loi and Molinari, 2020).

### RTN3

RTNs are a family of ER-resident proteins that contain RHDs, and four subtypes have been identified: RTN1–4 (Yan et al., 2006). The oligomerization of the long isoform of RTN3 promotes ER fragmentation; RTN3L, a long isoform of RTN3, interacts with LC3 *via* the LIR, and the fragmented ER is eventually transferred to lysosomes (Grumati et al., 2017). Although both FAM134B and RTN3 are key proteins mediating ER-phagy, they are localized at different sites of the ER. RTN3 is mainly located in ER tubules, while FAM134B resides in ER sheets. In addition, these proteins do not interact, and FAM134B and RTN3 mediate ER-phagy independently (Grumati et al., 2017).

### CCPG1

Cell cycle progression gene 1 (CCPG1) is a single-pass transmembrane protein located in the ER within the lumen and cytosolic regions. The transcription of CCPG1 increases upon the induction of ER stress *in vitro*, suggesting that CCPG1 is involved in maintaining ER homeostasis (Smith and Wilkinson, 2018). Consistent with this finding, the absence of CCPG1 results in the expansion and disruption of the ER as well as in the increased levels of ER stress indicators (Smith and Wilkinson, 2018). CCPG1 was shown to be involved in ER-phagy upon the screening of GABARAP-interacting proteins by affinity-mass spectrometry (Smith et al., 2018). Similar to other ER-phagy receptors, CCPG1 binds to ATG8 family proteins through the cytoplasmic region of the LIR motif, thus mediating the occurrence of ER-phagy. In addition, it also directly interacts with FIP2000, another critical protein in the autophagy pathway (Smith et al., 2018).

### TEX264

Testis expressed gene 264 (TEX264) is a single-channel transmembrane protein located throughout the entire ER network that contains an N-terminal hydrophobic region, a cytosolic gyrase inhibitor (GyrI)-like domain, and a C-terminal unstructured intrinsically disordered region (IDR) (An et al., 2019; Delorme-Axford et al., 2019). Through differential interactome screening using wild-type LC3B, TEX264 was identified as an ER-phagy receptor (Chino et al., 2019). Similarly, An et al. (2019) also identified TEX264 as an ER-phagy receptor by performing quantitative proteomic analysis under nutrient stress conditions. ER-phagy was substantially inhibited after TEX264 inhibition, and IP analysis showed that TEX264 had a stronger binding affinity for LC3 and GABARAP family proteins than for the other four ER-phagy receptors (FAM134B, SEC62, RTN3L, and CCPG1) (Chino et al., 2019). ER-phagy mediated by TEX264 requires the participation of typical autophagy pathway components, such as ATG8 family proteins and the class III phosphatidylinositol 3-kinase complex (An et al., 2019).

### ATL3

Atlastins (ATLs) are membrane-bound GTPases that participate in the regulation of ER shape, and three ATL subtypes are expressed in humans: ATL1, ATL2, and ATL3 (Zhao et al., 2016). ATL1 is mainly expressed in the central nervous system, while ATL2 and ATL3 are more widely distributed (Rismanchi et al., 2008). Recently, Chen et al. reported ATL3 to be an ER-phagy receptor that binds specifically to GABARAPs through two GABARAP-interacting motifs (GIMs). ATL3 mediates the degradation of tubular ER under starvation conditions (Chen et al., 2019). Both RTN3 and ATL3 are tubular ER-phagy receptors, which raises the question of why do two receptors mediate the selective autophagy of tubular ER. One potential explanation is that they may be involved in ER-phagy in different cells due to the differences between tissues and cell types. Moreover, they may work together to regulate the degradation of tubular ER.

## Other Receptors in Mammals

BCL2/adenovirus E1B 19-kDa protein-interacting protein 3 (BNIP3) is located in the outer mitochondrial membrane and has been shown to be involved in mitophagy (Zhang and Ney, 2009; O’Sullivan et al., 2015). BNIP3 directly binds to LC3 through its LIRs, thus facilitating the clearance of damaged mitochondria (Ney, 2015; Tang et al., 2019). However, BNIP3 has also been detected on the ER (Zhang et al., 2009, 2010), and Bozi et al. (2018) observed increased BNIP3 expression in cells subjected to ER stress. These findings led to the speculation that BNIP3 functions as a receptor for ER-phagy. In another recent study, CALCOCO1 was identified as a soluble ER-phagy receptor for the degradation of tubular ER. Mechanistically, CALCOCO1 binds to ER proteins (VAPA and VAPB) through its FFAT-like motif, but it also recruits ATG8 through its LIR and UDS-interacting region (UIR) motifs to trigger ER-phagy (Nthiga et al.,2020a,b).

In general, the stimulation of ER stress and nutrient deficiency and other factors lead to changes in the expression levels of ER-phagy receptors, which mediate ER-phagy in different ER subdomains and different situations, thereby resulting in excessive ER clearance in a timely manner to maintain cellular stability in mammals.

## ER-Phagy Receptors in Yeast

### ATG39 and ATG40

**FIGURE 3:**
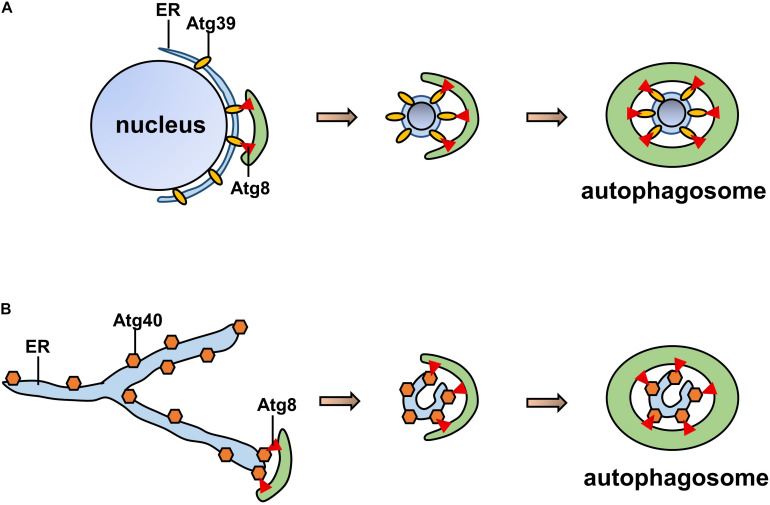
Pattern of ATG39/40-dependent ER-phagy. **(A)** ATG39-dependent ER-phagy of the perinuclear ER. **(B)** ATG40-dependent cortical and cytoplasmic ER-phagy. Both ATG39 and ATG40 contain AIMs that interact with ATG8 to form autophagosomes.

In *Saccharomyces cerevisiae*, ATG39 and ATG40 are the two proteins that mediate ER-phagy. The yeast ER consists of the cytoplasmic ER (cytoER), cortical ER (cER), and perinuclear ER (pnER). ATG39 is involved in ER-phagy in the perinuclear ER, while ATG40 participates in cortical and cytoplasmic ER-phagy (Nakatogawa and Mochida, 2015). Thus, two ER-phagy receptors degrade different ER subdomains, and their functions are similar to that of the mammalian receptor RETREG1/FAM134B (Mochida et al., 2015). Both ATG39 and ATG40 contain ATG8-interacting motifs (AIMs), *via* which they interact with ATG8 to form autophagosomes (Fregno and Molinari, 2018). ATG40-mediated ER-phagy depends on the reticular structure of the ER, which forms highly curved regions that fuse with the autophagosome (Mochida et al., 2020); ATG39-mediated ER-phagy is also regarded as “nucleophagy” due to the double-membrane vesicles that encapsulate nuclear proteins (Nakatogawa, 2020; Figure 3). Similar to other autophagy processes, ER-phagy in yeast is regulated by many factors. In nutrient-sufficient conditions, ATG39 and ATG40 are repressed, while their expression levels are increased in the absence of nitrogen sources. In addition, intervention with rapamycin increased the expression levels of ATG39 and ATG40 (Mochida et al., 2015; Nakatogawa and Mochida, 2015).

### ERP1

Epr1 is a new receptor that has been indicated to be involved in ER-phagy in *Schizosaccharomyces pombe*. Similar to other ER-phagy receptors, Epr1 interacts with ATG8 directly through its AIM region. Interestingly, unlike transmembrane proteins in mammals, Epr1 is a soluble protein that is localized in the ER through its interaction with the ER membrane proteins VAPs (Zhao et al., 2020). Briefly, Epr1 serves as a bridge between VAPs and the autophagy-related protein ATG8, and its effect is substituted by an artificial ATG8-VAP tether (Zhao and Du, 2020; Zhao et al., 2020).

## ER-Phagy Receptor in Plants

The first ER-phagy receptors to be identified in plants were ATI1 and ATI2, which contain a single transmembrane domain and an AIM at the N-terminus in the cytoplasm (Honig et al., 2012). After carbon starvation, the proteins localize in ER-associated bodies and are subsequently transported to vacuoles (Michaeli et al., 2014) once they interact with ATG8f (Honig et al., 2012). In addition, Arabidopsis Sec62 (AtSec62) is an essential protein for plant development that may function as an ER-phagy receptor (Hu et al., 2020). Mutation of AtSec62 stunts plant growth and increases its sensitivity to tunicamycin (TM)-induced ER stress, while overexpression of AtSec62 increases resistance to ER stress and is accompanied by increased colocalization with ATG8 (Hu et al., 2020). Moreover, Rtn1 and Rtn2 interact with ATG8a though four AIMs, and the binding of Rtn2 to ATG8 is increased in response to ER stress (Zhang et al., 2020). In addition, C53 is a soluble protein found in plants and mammals that was identified as an ER-phagy receptor through a peptide-competition assay coupled with an affinity proteomics screen (Stephani et al., 2020). C53 interacts with ATG8 *via* a shuffled AIM (a non-canonical AIM) under ER stress conditions and mediates autophagy upon the induction of ribosome stalling, leading to the degradation of ER proteins (Stephani et al., 2020).

## ER-Phagy and Disease

As ER-phagy is a key biological process in maintaining ER homeostasis, abnormal ER-phagy leads to the occurrence of diseases. However, as the study of ER-phagy is still in its infancy, and the receptors of ER-phagy are still being identified, few studies have directly linked ER-phagy with disease. Here, we will summarize some of the existing studies on the possible relationship between ER-phagy and diseases.

## Metabolic Diseases

FAM134B, one of the earliest identified ER-phagy receptors in mammals, has been shown to be involved in adipocyte differentiation. Mice overexpressing FAM134B in adipocytes show increased white adipose tissue (WAT) and obesity as well as high blood glucose levels and severe insulin resistance (Cai et al., 2019b). However, further mechanistic studies found that the effect of FAM134B on metabolism appears to be caused by its promotion of mitophagy rather than ER-phagy. Overexpression of FAM134B in three T3-L1 preadipocytes results in increased autophagy, decreased mitochondrial numbers, and the promotion of differentiation, and these phenomena are inhibited by treatment with an autophagy inhibitor (3-methyladenine) (Cai et al., 2019a). ARF-related protein 1 (ARFRP1) is involved in protein trafficking (Werno et al., 2018), and Cai et al. demonstrated that FAM134B increases the expression of ARFRP1, which promotes lipid accumulation (Cai et al., 2019a). Similarly, another ER-phagy receptor, RTN3, was reported to participate in lipid accumulation, and increased expression of RTN3 was observed in obese and hypertriglyceridemic patients. Moreover, mice overexpressing RTN3 showed obesity and higher levels of triglycerides (Xiang et al., 2018). Furthermore, by interacting with heat shock protein family A (Hsp70) member 5 (HSPA5), RTN3 activates two important downstream molecules that regulate triglyceride biosynthesis, SREBP-1c and AMPK (Xiang et al., 2018). In addition, RTN3 controls the secretion of very low-density lipoprotein (VLDL) by regulating VLDL transport vesicles (Siddiqi et al., 2018).

## Neurological Diseases

Noticeable increases in autophagy levels, decreases in the ER Ca^2+^ concentration, and increases in ROS levels are observed in hippocampal neuronal culture models of acquired epilepsy (AE), while the upregulation of FAM134B expression reverses these changes (Xie et al., 2020). In addition, a missense mutation of ATL3 (p. Tyr192Cys and P338R) was identified in a family with sensory neuropathy and loss of pain perception through whole-exome sequencing (Fischer et al., 2014; Kornak et al., 2014). The most direct evidence of the relationship between ER-phagy and nervous system diseases was provided by Chen et al. (2019) who showed that these two mutations significantly abolished the interaction between ATL3 and GABARAP, thus abrogating the re-establishment of ER-phagy. Similarly, mutations in FAM134B are the cause of hereditary sensory and autonomic neuropathy type II (Kurth et al., 2009). Further supporting the speculation that abnormal ER-phagy is involved in the development of neurological diseases is that RTN3-immunoreactive dystrophic neurites (RIDNs) and the accumulation of high-molecular-weight RTN3 in patients with AD cases and mouse models result in neuronal dystrophy, which eventually leads to impairments in spatial learning and memory (Hu et al., 2007).

## Other Diseases

In addition to the metabolic and neurological diseases mentioned above, ER-phagy may also play a role in other diseases. The absence of FAM134B results in an increase in the production of infectious Ebola virus (EBOV) by 1- to 2-log10-fold (Chiramel et al., 2016), suggesting that the ER-phagy mediated by FAM134B is a limiting event for EBOV infection. Moreover, CCPG1-dependent ER-phagy maintains pancreatic homeostasis *in vivo* through the timely removal of insoluble proteins from the ER, thus preventing the occurrence of pancreatitis(Smith et al., 2018).

## Conclusion

**FIGURE 4:**
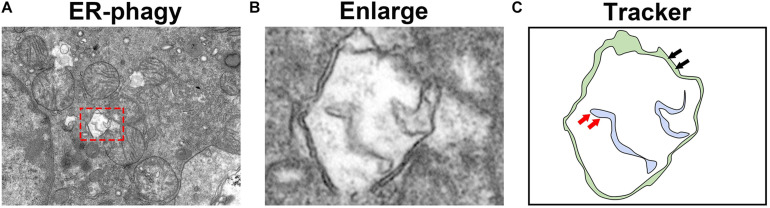
Observation of ER-phagy by transmission electron microscopy. **(A)** Transmission electron microscopy images of the mouse kidney. **(B)** The autophagosomes involved in ER-phagy. **(C)** The localization pattern of autophagosomes in ER-phagy (the red arrow represents the ER membrane, and the black arrow represents the autophagosome membrane).

As the ER is one of the most important cellular organelles, ER homeostasis is essential for all life activities. ER-phagy protects cells from excessive ER stress by removing the damaged ER in a timely manner (Figure 4). During the UPR, the ER is expanded to counteract cellular stress. Once the stress stimulus subsides, the excess ER generated during the acute UPR phase is removed by receptor-mediated ER-phagy. Recently, a series of new ER-phagy receptors have been identified, and abnormal ER-phagy mediated by these receptors plays an important role in diseases. Therefore, the artificial regulation of ER-phagy will be a therapeutic strategy for some diseases in the future. However, unlike ER stress, our understanding of ER-phagy remains in its infancy, and further research on its relationship with diseases and its underlying molecular mechanisms are needed. Moreover, whether specific ER-phagy activators or inhibitors exist and which type of receptor-mediated ER-phagy plays the most important role in disease development need to be elucidated. Although substantially more research is needed, better comprehension of ER-phagy will further our understanding of the pathogenesis of some diseases. Moreover, as a new therapeutic target, ER-phagy still has invaluable significance in future research.

## Author Contributions

MY, SL, XW, CL, and JY designed and performed the study. XZ and LX conceived the project. MY and LS wrote the manuscript. All authors contributed to the article and approved the submitted version.

## Conflict of Interest

The authors declare that the research was conducted in the absence of any commercial or financial relationships that could be construed as a potential conflict of interest.
